# The Synergistic Effects of Low Dose Fluorouracil and TRAIL on TRAIL-Resistant Human Gastric Adenocarcinoma AGS Cells

**DOI:** 10.1155/2013/293874

**Published:** 2013-11-13

**Authors:** Hong Zhu, Min Huang, Daoling Ren, Jianping He, Fen Zhao, Cheng Yi, Ying Huang

**Affiliations:** ^1^Department of Abdominal Cancer, West China Hospital, Sichuan University, Chengdu, Sichuan 610041, China; ^2^Department of Pathophysiology, West China School of Preclinical and Forensic Medicine, Sichuan University, Chengdu, Sichuan 610041, China; ^3^Department of Nuclear Medicine, Tai'an Central Hospital, Tai'an, Shandong 271000, China

## Abstract

The TNF-related apoptosis-inducing ligand (TRAIL) is a TNF family member which has been under intense focus because of its remarkable ability to induce apoptosis in malignant human cells while leaving normal cells unscathed. However, many cancer cells remain resistant to TRAIL. In this study, we had investigated the synergistic effects of low dose fluorouracil (5-Fu) and TRAIL on TRAIL-resistant human gastric adenocarcinoma AGS cells and explored the potential mechanisms. Cell viability was analyzed by sulforhodamine B (SRB) assay and the synergistic effects were evaluated by Jin's formula and confirmed by both morphological changes under inverted microscope and flow cytometry. The expression of TRAIL-R1 (death receptor 4, DR4), TRAIL-R2 (DR5), TRAIL-R3 (decoy receptor, DcR1), TRAIL-R4 (DcR2), procaspase-3, procaspase-8, and procaspase-9 was detected by western blotting. Our results showed that there were significant synergistic effects of low dose 5-Fu and TRAIL on TRAIL-resistant AGS cells, and this effect was supposed to be mediated by decreasing DcR2 expression and increasing DR5 expression. The extrinsic and intrinsic apoptosis pathways were both activated. The data suggest that combined treatment of low dose 5-Fu and TRAIL can be an effective therapeutic approach for gastric adenocarcinoma.

## 1. Introduction 

 The TNF-related apoptosis-inducing ligand (TRAIL) is a TNF family member capable of inducing apoptosis through caspase-dependent mechanisms. TRAIL can bind to 5 different receptors: TRAIL-R1 (death receptor 4, DR4), TRAIL-R2 (DR5), TRAIL-R3 (decoy receptor, DcR1), TRAIL-R4 (DcR2), and osteoprotegerin (OPG) [1]. DR4 and DR5 are the death receptors that signal for apoptosis, whereas DcR1 and DcR2 do not have the intracytoplasmic death domain to transduce apoptotic death signals, and thus they protect cells from TRAIL-mediated cell death by interfering with signaling through DR4 and DR5. Another receptor, osteoprotegerin (OPG), is a soluble receptor that may play a more prominent role in bone and myeloid cell development [2, 3]. TRAIL induces apoptosis in a wide variety of tumor cells but does not cause toxicity in most normal cells for the large numbers of decoy receptors on normal cells [4, 5]. The* in vivo* administration of TRAIL has been proved to be safe, unlike the other members of the TNF superfamily [6]. Thus, TRAIL is a promising cancer therapeutic agent due to its tumor selectivity. However, recent studies showed that many types of cancer cells have intrinsic or acquired resistance to TRAIL-induced apoptosis [5, 7, 8], which potentially restricts its use in treatment. Therefore, for the clinical use of TRAIL in cancer therapy, it is extremely important to overcome TRAIL resistance.

AGS cell lines have been shown to grow in athymic mice and to have the same histochemical and cytological characteristics as the specimen taken from the patient [9, 10]. So, it is important to characterize human tumor cells *in vitro*, and recently this cell line has been widely used as a model system for evaluating cancer cell apoptosis [11]. However, many studies found that AGS cell line acquired resistance to the apoptotic effects of TRAIL [9, 12–14].

Until now, there were some studies reported that natural products including some of their extracts such as *Pongamia pinnata* [15], cryptolepine [16], *Amoora cucullata* [6], sanguinarine [13], and genistein [9] could sensitize or overcome the resistance of AGS cells to TRAIL. However, no chemotherapy drug was reported. Fluorouracil (5-Fu) is considered as a cornerstone of therapy for patients with gastric cancer [17]. Past studies indicated that 5-Fu could enhance the apoptosis effects of TRAIL in some cancer cells such as hepatocellular carcinoma cells [18] and renal cell carcinoma cells [19]. Nevertheless, whether 5-Fu, especially when used in low dose, could increase the antitumor effects of TRAIL on TRAIL-resistant human gastric adenocarcinoma AGS cell was unknown. So, we carried out this study to investigate the combined effects of low dose 5-Fu and TRAIL on AGS cells and explore the potential mechanisms.

## 2. Materials and Methods 

### 2.1. Materials

Human gastric adenocarcinoma cell line AGS and TRAIL were provided by DIAO Group (China). 5-Fu was purchased from KINGYORK Co. (Tianjin, China). Sulforhodamine B (SRB) was supplied by Sigma (USA). Roswell Park Memorial Institute (RPMI) 1640 medium and trypsin were purchased from Gibco (USA). Mouse monoclonal antibody for procaspase-3, DR5, and *β*-actin and rabbit monoclonal antibody for procaspase-8, DcR2, and DR4 were purchased from Santa Cruz Biotechnology (USA). Rabbit monoclonal antibody for procaspase-9 was purchased from Lab Vision (USA). Rabbit monoclonal antibody for DcR1 was purchased from AnaSpec Co. (USA). Goat anti-mouse IgG-HRP and goat anti-rabbit IgG-HRP were purchased from KPL Co. (USA). Polyvinylidene difluoride (PVDF) membrane was purchased from Millipore (USA). Enhanced chemiluminescence (ECL) detection kit was purchased from Roche (Swiss). Annexin V-FITC apoptosis detection kit was purchased from KeyGEN Biotech Co., Ltd (China).

### 2.2. Cell Culture

The human gastric adenocarcinoma cell line AGS was cultured in RPMI 1640 medium supplemented with 10% fetal bovine serum plus ampicillin and streptomycin routinely and incubated in 5% CO_2_ at 37°C.

### 2.3. TRAIL and Fluorouracil-Mediated Toxicity Evaluated by SRB Methods

The cell inhibition rates of TRAIL and 5-Fu were measured by SRB method [5]. Exponentially growing tumor cells were seeded into a 96-well plate (1 × 10^5^/well), and then TRAIL or 5-Fu was added to each well 24 h after incubation, respectively. The final concentrations of TRAIL were 0.0461, 0.137, 0.412, 1.953, 7.812, 31.25, 125, 250, 500, and 1000 *μ*g/mL, respectively. The final concentrations of 5-Fu were 0.03, 0.09, 0.27, 0.82, 2.47, 7.41, 22.2, 66.7, and 200 *μ*g/mL, respectively. The cytotoxic effect was evaluated 48 h after drug challenge. Fifty *μ*L of 50% trichloroacetic acid was added and incubated for 60 min at 4°C. After washing and drying the plate, 50 *μ*L of 0.4% SRB was added for 20 min. The plates were rinsed with 0.1% acetic acid and air-dried, after which 200 *μ*L of tris base (10 mmol/L) was added, and the plates were shaken for 5 min. The SRB value was measured at a wavelength of 490 nm. All SRB experiments were performed in triplicate and repeated at least three times.

The dose response curves were depicted applying OriginPro 7.5 software. The concentrations of reagents that induced a 50% reduction in cell viability (IC_50_) were determined from the curves of reagent concentration versus cell inhibition rate at 48 h of incubation for the cell line analyzed. The sensitivity of cells to drug is evaluated by the value of IC_50_. IC_50_ < 10 *μ*g/mL, which indicates that cells are sensitive to drug, while IC_50_ ≥ 10 *μ*g/mL, which suggests that cells are relatively resistant to drug [20].

### 2.4. Evaluation of Synergetic Effect by Jin's Formula

According to the effect of TRAIL and 5-Fu treatment alone on cell viability, the concentrations of the combination of TRAIL and 5-Fu were chosen as follows: 0.4, 2, 10, and 50 *μ*g/mL, respectively of TRAIL and 0.05, 0.1, 0.5, 1, and 5 *μ*g/mL, respectively of 5-Fu. Cell inhibition rates were then assessed by SRB assay. Synergetic effect of the combination of TRAIL and 5-Fu was analyzed by Jin's formula [21–23]. The formula is *Q* = *Ea* + *b*/(*Ea* + *Eb* − *Ea* × *Eb*), where *Ea* + *b*, *Ea* and *Eb* are the average effects (inhibition rate) of the combination treatment, 5-Fu only, and TRAIL only, respectively. In this method, *Q* < 0.85 indicates antagonism, 0.85 ≤ *Q* < 1.15 indicates additive effects, and *Q* ≥ 1.15 indicates synergism. According to the results of synergetic effect, the concentrations of 2 *μ*g/mL TRAIL and 0.5 *μ*g/mL 5-Fu (low dose) were chosen for later experiments.

### 2.5. Morphological Changes under Inverted Microscope

The AGS cells were counted and cultured in 6-well plates at a concentration of 2 × 10^5^ cells per well. Twenty four h after the incubation, they were then divided into four groups: the control group treated with normal saline; the 5-Fu group treated with 0.5 *μ*g/mL 5-Fu; the TRAIL group treated with 2 *μ*g/mL TRAIL; and the combined group treated with 0.5 *μ*g/mL 5-Fu and 2 *μ*g/mL TRAIL. Twenty four h after the treatment, the morphological changes of cells in each group were observed under an inverted microscope (Olympus, Japan).

### 2.6. Flow Cytometry Analysis

 Exponentially growing tumor cells were seeded in cell culture flask (75 mL) with a concentration of 1 × 10^5^/mL, 10 mL per flask. Twenty four h after incubation, they were then divided into four groups: the control group treated with saline; the 5-Fu group treated with 0.5 *μ*g/mL 5-Fu; the TRAIL group treated with 2 *μ*g/mL TRAIL; and the combined group treated with 0.5 *μ*g/mL 5-Fu and 2 *μ*g/mL TRAIL. Twenty four h and 48 h after the treatment, the cells were subjected to flow cytometry using Annexin V-FITC apoptosis detection kit following the manufacturer's instruction. A minimum of 10^4^ cells were analyzed in each sample. All experiments were repeated at least 3 times.

### 2.7. Western Blot Analysis

Cells incubated with 0.5 *μ*g/mL 5-Fu or 2 *μ*g/mL TRAIL alone or in combination for 48 h were lysed in lysis buffer. Protein content of the supernatant was measured using bicinchoninic acid (BCA) method [24]. Fifteen *μ*g of cell lysate protein was separated by SDS-PAGE using a Tris-glycine system and then the gels were electroblotted onto PVDF membranes for 45 to 60 min. The membranes were then incubated with 5% nonfat dry milk in PBS for 1 h for blocking the nonspecific binding sites and then incubated with the appropriate primary antibody concentration (1 : 750 dilution for DcR1; 1 : 400 dilution for DcR2 and DR4; 1 : 500 dilution for DR5; 1 : 500 dilution for procaspase-3, procaspase-8, and procaspase-9; and 1 : 2000 for *β*-actin) overnight at 4°C in 5% nonfat dry milk. Membranes were subsequently rinsed in PBS and then incubated at 37°C with secondary antibody (1 : 3000 dilutions). After the membranes were exposed to the respective secondary antibodies for 2 hours, the blots were analyzed through chemiluminescence detection and autoradiography.

### 2.8. Statistical Analysis

The results were expressed as the mean ± standard deviation (SD). Statistical comparisons of mean values were analyzed by one-way ANOVA using SPSS 16.0 software. The dose response curves were depicted applying OriginPro 7.5 software. The synergistic effects were evaluated by Jin's formula. All *P* values were two-sided and *P* < 0.05 was considered as statistically significant.

## 3. Results 

### 3.1. SRB Assay

**Figure 1:**
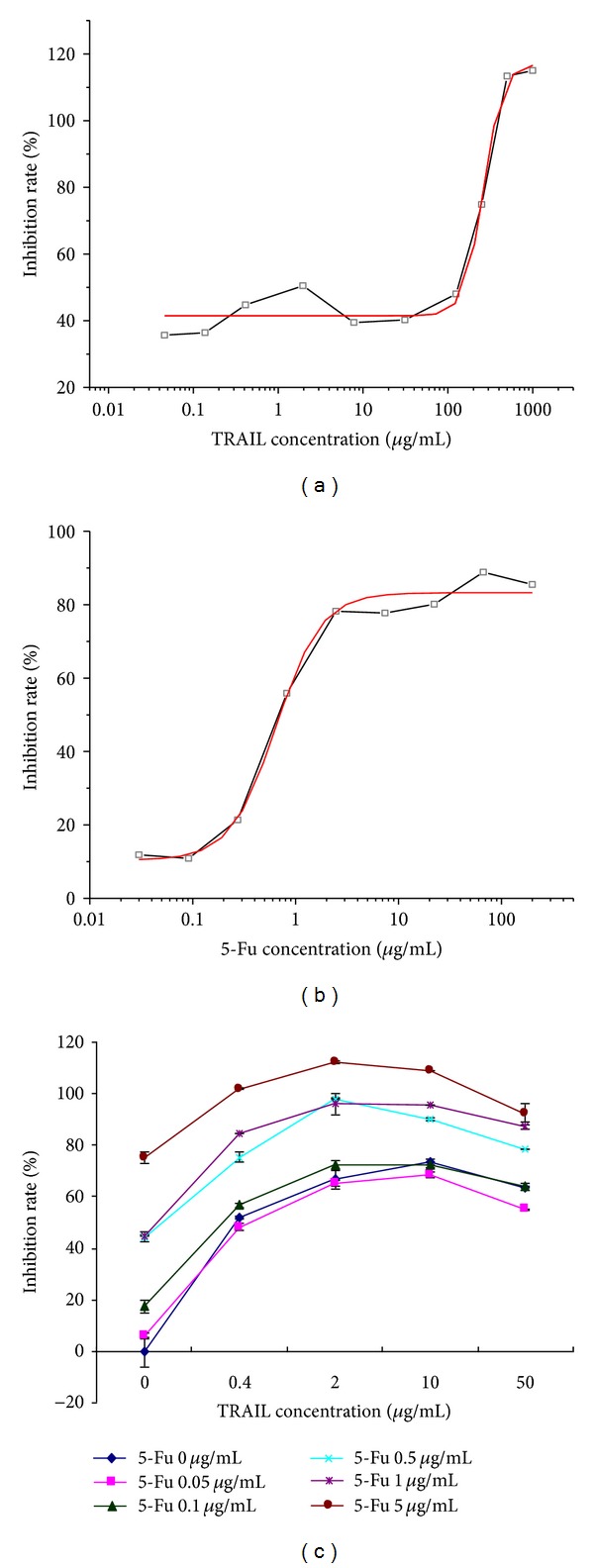
SRB assay. (a) TRAIL treatment; (b) 5-Fu treatment; and (c) combination treatment. The dose response curves were depicted applying OriginPro 7.5 software (*R* ^2^ = 0.979 for TRAIL; *R* ^2^ = 0.992 for 5-Fu). There was significant positive correlation between cell inhibitive rate and TRAIL concentration between 0 *μ*g/mL and 1000 *μ*g/mL (*r* = 0.921, *P* < 0.01). IC_50_ was 261.60 ± 23.38 *μ*g/mL which was larger than 10 *μ*g/mL suggesting that AGS cells were relatively resistant to TRAIL. There was significant positive correlation between cell inhibitive rate and 5-Fu concentration between 0 *μ*g/mL and 100 *μ*g/mL (*r* = 0.735, *P* < 0.05). IC_50_ was 0.646 ± 0.078 which was lower than 10 *μ*g/mL suggesting that AGS cells were sensitive to 5-Fu.

 As shown in Figure 1, the dose response curves were depicted applying OriginPro 7.5 software (*R* ^2^ = 0.979 for TRAIL; *R* ^2^ = 0.992 for 5-Fu). There was significant positive correlation between cell inhibition rate and TRAIL concentration between 0 *μ*g/mL and 1000 *μ*g/mL (*r* = 0.921, *P* < 0.01). IC_50_ was 261.60 ± 23.38 *μ*g/mL which was larger than 10 *μ*g/mL suggesting that AGS cells were relatively resistant to TRAIL (Figure 1(a)). There was significant positive correlation between cell inhibition rate and 5-Fu concentration between 0 *μ*g/mL and 100 *μ*g/mL (*r* = 0.735, *P* < 0.05). IC_50_ was 0.646 ± 0.078 which was lower than 10 *μ*g/mL indicating that AGS cells were sensitive to 5-Fu (Figure 1(b)). According to the results of Jin's formula, six combination models showed synergism on AGS cells (Figure 1(c), Table 1). The concentrations of 2 *μ*g/mL TRAIL and 0.5 *μ*g/mL 5-Fu (low dose) were chosen to investigate the combined effects of low dose 5-Fu and TRAIL on TRAIL-resistant AGS cells and explore the potential mechanisms.

### 3.2. The Morphological Changes under Inverted Microscope

**Figure 2:**
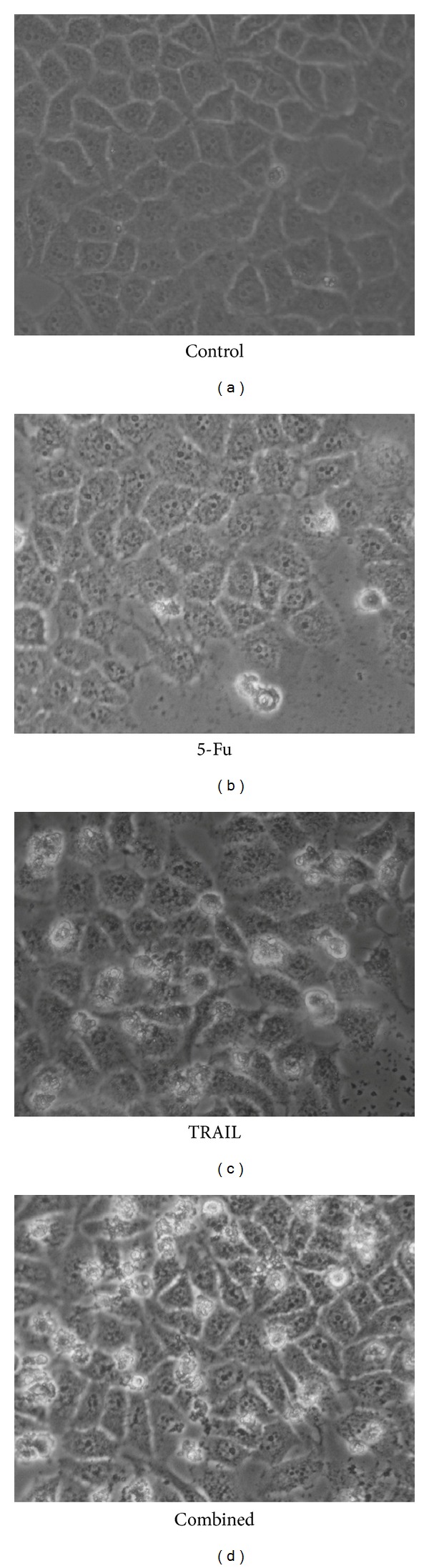
Morphological changes in the AGS cells under an inverted microscope after 24 h of treatment (400x). Control: AGS cells treated with saline; 5-Fu: AGS cells treated with 0.5 *μ*g/mL 5-Fu; TRAIL: AGS cells treated with 2 *μ*g/mL TRAIL; combined: AGS cells treated with 0.5 *μ*g/mL 5-Fu and 2 *μ*g/mL TRAIL.

As shown in Figure 2, 24 h after the treatment, the cells of both 5-Fu and TRAIL groups showed evident apoptosis, and the apoptosis was more remarkable in the combined group. In the combined group, most of cells were floated on the supernatant and only a few cells grew along the wall. The cells underwent significant changes in morphology; their original shape was completely altered, the cytoplasm became rougher, the nucleus became pycnotic, and the refractive index in the cells decreased, demonstrating significant cellular damages. By contrast, AGS cells in the control group did not present significant morphological changes.

### 3.3. Flow Cytometry

**Figure 3:**
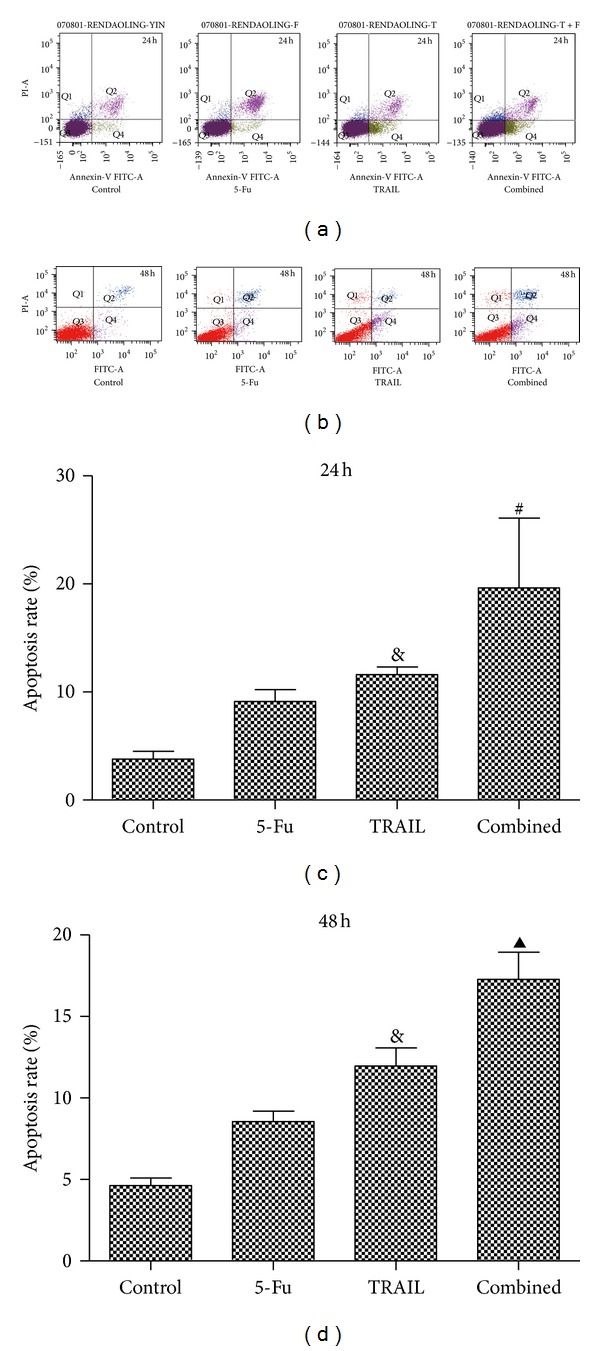
Flow cytometry. (a) Flow cytometry 24 h after treatment; (b) flow cytometry 48 h after treatment; (c) histogram of flow cytometry 24 h after treatment; and (d) histogram of flow cytometry 48 h after treatment. Control: AGS cells treated with saline; 5-Fu: AGS cells treated with 0.5 *μ*g/mL 5-Fu; TRAIL: AGS cells treated with 2 *μ*g/mL TRAIL; and combined: AGS cells treated with 0.5 *μ*g/mL 5-Fu and 2 *μ*g/mL TRAIL. The apoptosis rates were 3.8 ± 0.7%, 9.1 ± 1.1%, 11.6 ± 0.7%, and 19.65 ± 6.45%, respectively, in the control, 5-Fu, TRAIL, and combined groups 24 h after treatment and were 4.63 ± 0.47%, 8.55 ± 0.65%, 11.96 ± 1.12%, and 17.27 ± 1.67%, respectively, in the control, 5-Fu, TRAIL, and combined groups 48 h after treatment. ^&^ *P* < 0.05 compared with control and 5-Fu groups, ^#^ *P* < 0.05 compared with control, 5-Fu, and TRAIL groups, and ^▲^ *P* < 0.01 compared with control, 5-Fu, and TRAIL groups.

The apoptosis rates were 3.8 ± 0.7%, 9.1 ± 1.1%, 11.6 ± 0.7%, and 19.65 ± 6.45%, respectively, in the control, 5-Fu, TRAIL, and combined groups 24 h after treatment (Figure 3(a)) and were 4.63 ± 0.47%, 8.55 ± 0.65%, 11.96 ± 1.12%, and 17.27 ± 1.67%, respectively, in the control, 5-Fu, TRAIL, and combined groups 48 h after treatment (Figure 3(b)). The apoptosis rates were higher in TRAIL group than control and 5-Fu groups at both 24 h and 48 h (*P* < 0.05). More important, there were significant differences of apoptosis rates in the combined group via other groups 24 h (*P* < 0.05) and 48 h (*P* < 0.01) after the treatment (Figures 3(c) and 3(d)).

### 3.4. DcR1, DcR2, DR4, and DR5 Expression

**Figure 4:**
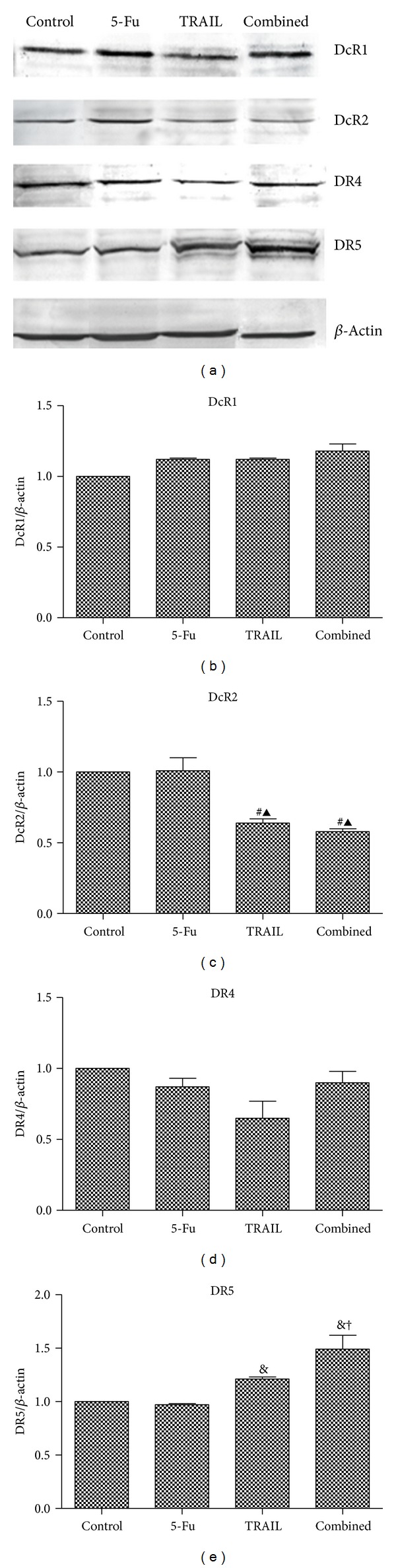
DcR1, DcR2, DR4, and DR5 expression. (a) Western blotting of DcR1, DcR2, DR4, and DR5; (b) histogram of DcR1 expression; (c) histogram of DcR2 expression; (d) histogram of DR4 expression; and (e) histogram of DR5 expression. Control: AGS cells treated with saline; 5-Fu: AGS cells treated with 0.5 *μ*g/mL 5-Fu; TRAIL: AGS cells treated with 2 *μ*g/mL TRAIL; combined: AGS cells treated with 0.5 *μ*g/mL 5-Fu and 2 *μ*g/mL TRAIL. ^#^ *P* < 0.01 compared with control group. ^▲^ *P* < 0.05 compared with 5-Fu group. ^&^ *P* < 0.01 compared with control and 5-Fu groups.^†^ *P* < 0.05 compared with TRAIL group.

The expression of TRAIL receptors was shown in Figure 4. From the results, we found that there was no statistical difference of DcR1 and DR4 expression in the four treatment groups in this study. However, the expression of DcR2 was remarkably decreased in the TRAIL and combined groups compared with the control (*P* < 0.01) and 5-Fu groups (*P* < 0.05). Besides, the expression of DR5 was significantly increased in the TRAIL and combined groups compared with the control (*P* < 0.01) and 5-Fu groups (*P* < 0.01), especially in the combined group (*P* < 0.05 versus TRAIL group).

### 3.5. Procaspase-3, Procaspase-8, and Procaspase-9 Expression

**Figure 5:**
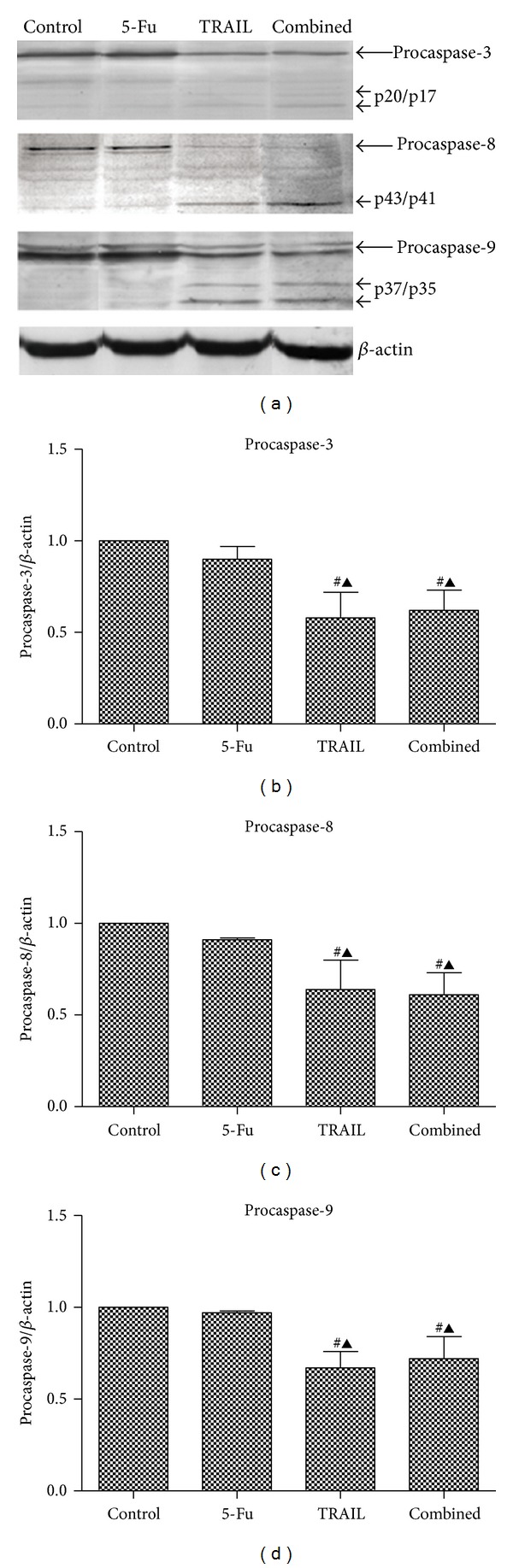
Procaspase-3, procaspase-8, and procaspase-9 expression. (a) Western blotting of procaspase-3, procaspase-8, and procaspase-9; (b) histogram of procaspase-3 expression; (c) histogram of procaspase-8 expression; (d) histogram of procaspase-9 expression. Control: AGS cells treated with saline; 5-Fu: AGS cells treated with 0.5 *μ*g/mL 5-Fu; TRAIL: AGS cells treated with 2 *μ*g/mL TRAIL; combined: AGS cells treated with 0.5 *μ*g/mL 5-Fu and 2 *μ*g/mL TRAIL. ^#^ *P* < 0.01 compared with control group. ^▲^ *P* < 0.05 compared with 5-Fu group.

As shown in Figure 5, the expression of procaspase-3, procaspase-8, and procaspase-9 in TRAIL and combined groups was significantly decreased compared with the control (*P* < 0.01) and 5-Fu groups (*P* < 0.05), indicating remarkable apoptosis after the treatment of TRAIL or the combination treatment of TRAIL and 5-Fu.

## 4. Discussion 

 Since its discovery in 1995, TRAIL, a member of the tumor necrosis factor superfamily, has been under intense focus because of its remarkable ability to induce apoptosis in malignant human cells while leaving normal cells unscathed [25]. So, it is safe and this was further confirmed by clinical trials [26, 27]. The majority of the studies published since the initial report describing TRAIL have focused on the *in vitro* and *in vivo* tumoricidal activity of TRAIL. In these experiments, TRAIL has induced apoptosis in multiple malignant cell lines, derived from both solid and hematologic malignancies [28–30]. However, TRAIL resistance is a major problem of its therapy, as a considerable number of cancer cells, especially some highly malignant tumors, are resistant to apoptosis induction by TRAIL [1, 7]. However, TRAIL remains a promising biologically targeted anticancer therapy that is currently in phase II clinical trials [26, 27, 31]. Researches over the last decade have exposed the complexity of TRAIL signaling and a myriad of resistance mechanisms to TRAIL-induced killing that are present in many human tumor cells [7]. Chemotherapy agents and radiotherapy appear to sensitize cells to the effects of TRAIL [25]. Thus, many tumors resistant to TRAIL-mediated apoptosis when TRAIL is used as a single agent will respond to a combination of conventional chemotherapy or radiotherapy plus TRAIL [5, 32, 33].

Gastric cancer remains a significant problem worldwide despite a declining incidence in the West. It remains the second most frequently diagnosed cancer worldwide, accounting for 9.9% of all new cancer diagnoses and responsible for 12.1% of all cancer deaths [34]. Since INT0116 trail [35], postoperative chemoradiotherapy became the trend of treatment for gastric cancer, and chemotherapy regimens had renewed constantly to enhance treatment efficacy and decrease side effects. And 5-Fu, either itself or other advanced forms, was nearly an irreplaceable chemotherapy agent for gastric cancer [17, 36, 37]. However, 5-Fu is a chemotherapy drug which may cause serious side effects, so it is of great significance to find whether the combined use of low dose 5-Fu and TRAIL is effective on TRAIL-resistant gastric cancer cells. AGS cell is a human gastric adenocarcinoma cell line that was reported to be resistant to TRAIL. Although many natural products or their extracts were found to be able to enhance the antitumor effects of AGS cells to TRAIL [6, 12, 15], no chemotherapy agent was reported. So, it is our purpose to find whether the combined treatment of low dose 5-Fu and TRAIL could increase the treatment efficacy of AGS cells.

In this study, we further proved that AGS cells were resistant to TRAIL while sensitive to 5-Fu by SRB assay. The synergistic effects were evaluated by Jin's formula, and six combination models of 5-Fu and TRAIL showed synergy. As 5-Fu is a chemotherapy agent which has a higher toxicity than TRAIL, we chose the concentrations of 2 *μ*g/mL TRAIL and 0.5 *μ*g/mL 5-Fu (low dose) to investigate the combined effects of low dose 5-Fu and TRAIL on TRAIL-resistant AGS cells and explore the potential mechanisms. The morphological changes under inverted microscope and flow cytometry results either 24 h or 48 h after the treatments confirmed that low dose 5-Fu could significantly increase the treatment efficacy of TRAIL in TRAIL-resistant AGS cells. The apoptosis rates in the combined group were higher than 5-Fu or TRAIL treatment alone at both 24 h and 48 h after the treatment. So, the combined treatment of low dose 5-Fu and TRAIL showed potential prospect for gastric cancer.

In the past, many studies found that the sensitization of tumor cells to TRAIL was associated with upregulation of TRAIL death receptors (DR4, DR5) and downregulation of TRAIL decoy receptors (DcR1, DcR2) [7, 38–40]. To explore the potential mechanisms, we detected the expression of TRAIL receptors after the treatment. The data showed that the expression of DcR2 in the TRAIL and combined groups was significantly decreased compared with the control group and was more evident in the combined group (*P* > 0.05). What is more, the expression of DR5 was significantly increased in the TRAIL and combined groups, especially in the combined group (*P* < 0.05). However, there was no statistical difference for the expression of DcR1 or DR4 in all the treatment groups. So, we think that synergistic effects of TRAIL combined with low dose 5-Fu on AGS cells were mediated by decreasing DcR2 expression and increasing DR5 expression.

After the interaction of TRAIL with DR4 or DR5, the signals are transmitted into the cells through the functional cytoplasmic death domain which lead to the transformation of procaspase-8 into caspase-8 [1]. Activation of caspase-8 further leads to two different apoptotic pathways depending on the cell type. TRAIL induces apoptosis in a mitochondrial-independent manner (extrinsic), activating downstream effecter caspases such as caspase-3, whereas a mitochondrial-dependent pathway (intrinsic) proceeds via the activation of caspase-9, which then induces the execution phase of apoptosis [25]. In this study, the expression of procaspase-3, procaspase-8, and procaspase-9 was decreased in 5-Fu, TRAIL, and combined groups, especially in TRAIL and combined groups (*P* < 0.01) compared with control group. Our results indicated that combined treatment with low dose 5-Fu and TRAIL simultaneously activated extrinsic (caspase-8 and caspase-3) and intrinsic (caspase-9) pathways.

During the synergetic effect experiment, we found that the inhibition rate of 10 *μ*g/mL TRAIL alone was higher than 60% (Figure 1(c)), while the inhibition rate was about 40% calculated from the response curve of TRAIL on AGS cells (Figure 1(a)). In this study, we have used 0.0461, 0.137, 0.412, 1.953, 7.812, 31.25, 125, 250, 500, and 1000 *μ*g/mL of TRAIL alone for cytotoxicity analysis on AGS cells to evaluate the sensitivity of AGS cells to TRAIL. In fact, there were so many different concentrations between 0.0461 *μ*g/mL and 1000 *μ*g/mL which were not used during the experiment. As shown in Figure 1, although the distances between two different concentrations on abscissa were equal, the values differ significantly (e.g., 1–0.1 versus 1000–100). There were many different concentrations between two points on abscissa. Although the dose response curve could be depicted, it could not represent all the actual inhibition rates, it represents just a tendency. In fact, there could be a few big fluctuations of inhibition rates such as the concentration of 1.953 *μ*g/mL. So in Figure 1(a), 10 *μ*g/mL TRAIL is just a small point among the many different concentrations between 7.812 *μ*g/mL and 31.25 *μ*g/mL. There could be big fluctuation of inhibition rate at this point. What is more, we found that the expressions of procaspase-3, procaspase-8, and procaspase-9 were significantly decreased in the TRAIL alone and combined groups; however, the apoptosis rates were higher in combined group than TRAIL group. To explain the reasons, we think both the TRAIL alone group and combined group have induced apoptosis cascades; however, the apoptosis response was more significant in combined group. Besides, the 5-Fu in the combined group may induce apoptosis by other mechanisms.

In conclusion, the combined treatment of low dose 5-Fu and TRAIL showed significant synergistic effects on human gastric adenocarcinoma AGS cells and may be a promising treatment for gastric cancer. The effects were supposed to be mediated by decreasing DcR2 expression and increasing DR5 expression followed by an activation of both extrinsic and intrinsic apoptosis pathways.

## Figures and Tables

**Table 1:** Synergistic effect of TRAIL combined with 5-Fu on AGS cells analyzed by Jin's formula (*Q* value listed in the table).

5-Fu (*μ*g/mL)	TRAIL (*μ*g/mL)			
	0.4	2	10	50
0.05	0.88	0.95	0.91	0.84
0.1	0.94	0.99	0.92	0.91
0.5	1.03	1.20^#^	1.06	0.98
1	1.15^#^	1.17^#^	1.12	1.12
5	1.16^#^	1.22^#^	1.17^#^	1.02

According to Jin's formula, *Q* < 0.85 indicates antagonism, 0.85 ≤ *Q* < 1.15 indicates additive effects, and *Q* ≥ 1.15 indicates synergism (^#^). Synergism indicates that the effect of a mixture exceeds that expected from the individual components and additive effects (noninteraction) mean that the combined effect is equal to the expectation.
